# *Gliosarcoma *with Adenoid and* Chondrosarcomatous* Differentiation: A Case Report

**DOI:** 10.30699/ijp.2020.122606.2330

**Published:** 2020-10-10

**Authors:** Elham Jafari, Shiva Didehban, Shahriar Dabiri, Behshad Mofid

**Affiliations:** 1 *Pathology and Stem Cell Research Center, Kerman University of Medical Sciences, Kerman, Iran*; 2 *Clinical Research Unit, Shahid Bahonar Academic Center, Kerman University of Medical Sciences, Kerman, Iran*

**Keywords:** Adenoid Differentiation, Chondrosarcomatous Differentiation, Gliosarcoma

## Abstract

A heterogeneous group of CNS tumors are characterized by mixed neuroepithelial and mesenchymal features. Glial tumors manifesting this phenomenon are referred to as gliosarcoma. These tumors are usually mistaken for cerebral metastases or meningioma at operation. Their histological studies have revealed an admixture of gliomatous and sarcomatous tissues, which leads to a biphasic pattern. The mesenchymal component can present in different forms such as fibrosarcoma, undifferentiated pleomorphic sarcoma, chondro-osteogenic, and myogenic differentiation, as well as angiosarcomatous and liposarcomatous types. Squamous differentiation, *adenoid* formations and glandular structures may also be displayed.

Herein, we report a rare case who was admitted to the emergency room with decreased consciousness resembling methadone poisoning. Clinical work-up showed a temporoparietal mass on radiological investigation. Histopathological evaluation of the brain mass revealed a gliosarcoma with adenoid formations and a mesenchymal component, which manifested as chondrosarcomatous differentiation. Immunohistochemical studies confirmed the histologic diagnosis through positivity for EMA, GFAP, S100, and vimentin expression in different components.

## Introduction

Central nervous system tumors characterized by mixed neuroepithelial and mesenchymal features are highly heterogeneous. Most glial tumors manifesting mesenchymal features as sarcomatous elements are glioblastomas, so the designation of gliosarcoma is generally taken to connote a glioblastoma variant. According to the WHO 2016 classification of glial tumors, this tumor type is considered as grade IV. It has been suggested that approximately 2% of otherwise conventional glioblastomas generate malignant mesenchymal components. Clinically, they are divided into “primary” and “secondary” forms, based on whether they present as gliosarcoma de novo or the sarcomatous component only arises after the recurrence of a classic glioblastoma.

They usually affect the adult population in the fourth to the sixth decade of life. Men are more frequently affected than females (M: F ratio 1.8:1) (1, 2). 

Most of these tumors arise in the absence of recognized predisposing factors, but they have also been associated with prior irradiation, including the intracranial instillation of Thorotrast (1).

During operation, many are initially mistaken for cerebral metastases or (when attached to the dura) meningiomas, errors resulting from their characteristic circumscription and firm textures. These attributes in turn reflect the high content of connective tissue fibers typical of the gliosarcoma but foreign to most other neuroepithelial neoplasms (1).

Obviously, Gliosarcoma may be a poorly delineated peripheral grayish mass with central yellowish necrosis stippled with red and brown color change from recent and remote hemorrhage, while the sarcomatous component producing a firm discrete mass (2).

The strikingly biphasic architecture of these tumors observed in histological examination is due to a combination of gliomatous and sarcomatous tissues. The mesenchymal components would be quickly diagnosed as fibrosarcoma or undifferentiated pleomorphic sarcoma in a soft tissue setting, but chondro-osteogenic and myogenic differentiation may also be encountered in this setting. Variants that harbor angiosarcomatous, liposarcomatous, and mixed mesodermal-type elements have been reported as well. Squamous differentiation, adenoid formations, and glandular structures may also be displayed within the glial regions of selected cases (1).

The distinction of gliosarcoma’s dimorphic constituents can be further achieved using a combination of traditional histochemical and immunocytochemical techniques. Sarcomatous components are richly invested with connective tissue fibers, which can be observed through reticulin impregnation methods, but do not express GFAP, the reverse being true of the glial population (1).

We reported here a rare case of gliosarcoma that histologically presented chondrosarcomatous and adenoid formed components.

##  Case Report

A 53-year-old man with a history of opium addiction was admitted at the emergency ward with decreased level of consciousness (GCS=3) resembling methadone poisoning. Then, the patient was admitted to the Intensive Care Unit (ICU) and associated work-up such as brain CT scan was performed.

**Fig. 1:**
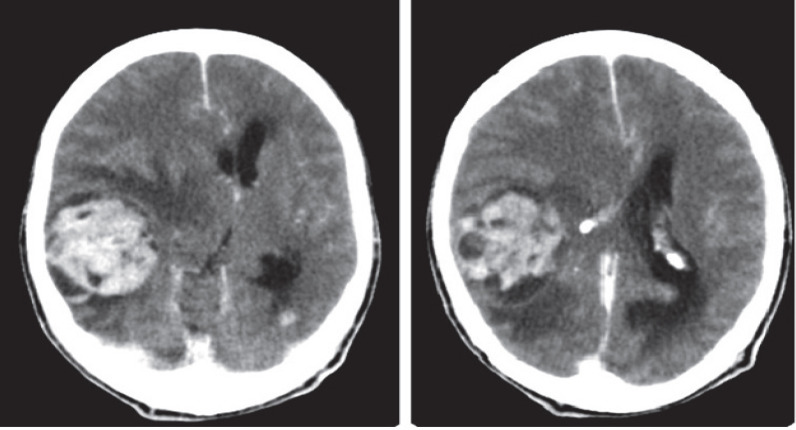
Heterodense mass with cystic and hemorrhagic components, severe peripheral edema and pressure effect in the right temporoparietal lobe

Spiral brain CT scan with IV contrast reported a 57×47 mm heterodense mass with cystic and hemorrhagic components and severe peripheral edema with pressure effect on the right lateral ventricle in the right temporoparietal lobe, which enhanced obviously with contrast. Midline shift to left side was also evident. As a result of imaging findings, the patient was transferred to the neurosurgery ward for surgical treatment (Figure 1).

Intraoperatively, an ill-defined lesion was seen with foci of hemorrhage suspicious for ICH or hemorrhagic brain tumor. Some parts of the mass had firm to hard consistency. Incisional biopsy was performed due to the large size of tumor and infiltrative borders.

**Fig. 2:**
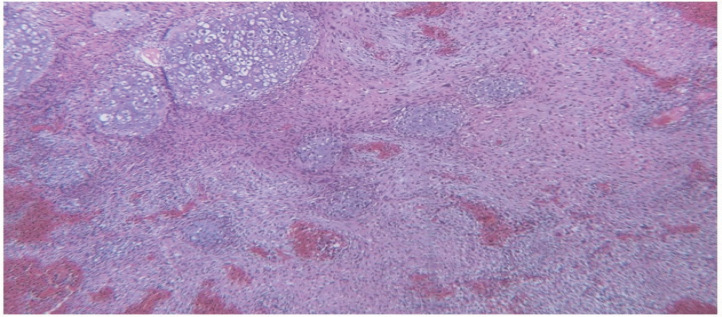
Mesenchymal component shows sarcomatous background with atypical cartilaginous areas (hematoxylin & eosin, 100× magnification)

**Fig. 3:**
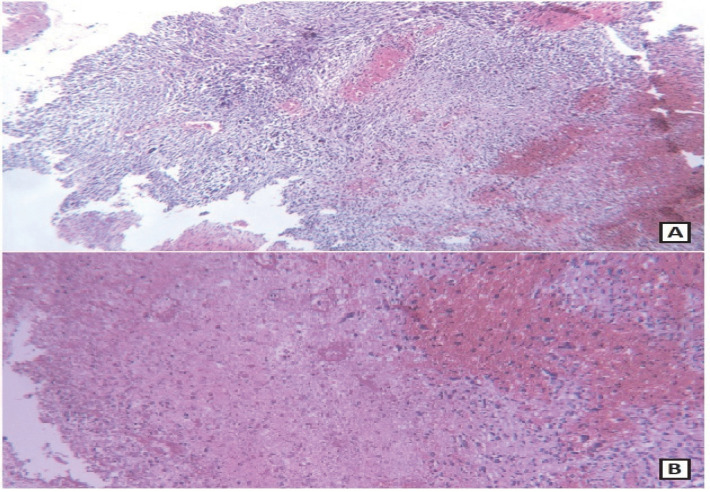
A, B: Glial region with necrosis aside spindle-shaped sarcomatoid component (A). (hematoxylin & eosin, A: 100× magnification, B: 400× magnification)

**Fig. 4:**
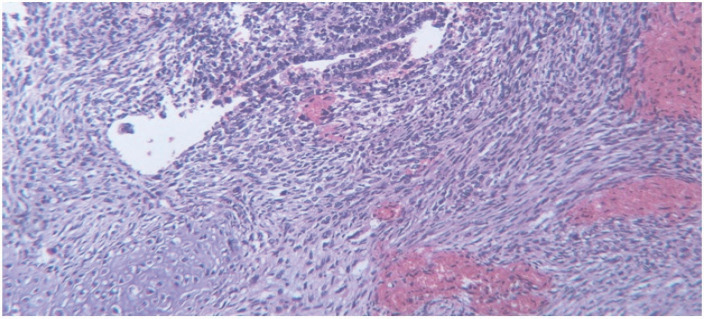
Adenoid formations in glial part surrounded by sarcomatoid areas (hematoxylin & eosin, 400× magnification)

The specimen received in the pathology ward consisting of multiple creamy brown fragments of brain tissue in total measuring 2.5×2×1.5 cm. The paraffin block and slides were prepared and stained with hematoxylin and eosin. On histological evaluation, brain tissue was seen with an infiltrative neoplastic growth, which showed a biphasic pattern, composed of malignant glial and mesenchymal components. The glial component showed increased cellularity with cells arranged in diffuse infiltrating sheets, having pleomorphic hyperchromatic nuclei arranged in fibrillary matrix.  Many cells with bizarre nuclear atypia interspersed with areas of pseudopalisading necrosis. Microvascular proliferation was not seen. The mesenchymal component contained spindle cells arranged in fascicles and sheets, along with nuclear atypia and mitoses. Areas of well-differentiated cartilaginous elements containing atypical chondrocytes with bizarre oval to spindle nuclei lying in lacunae and some of them containing more than one nuclei in one lacunae, surrounded by chondroid stroma, were seen. Some small areas also showed epithelial differentiation as adenoid formations (Figures 2-4)

**Fig. 5:**
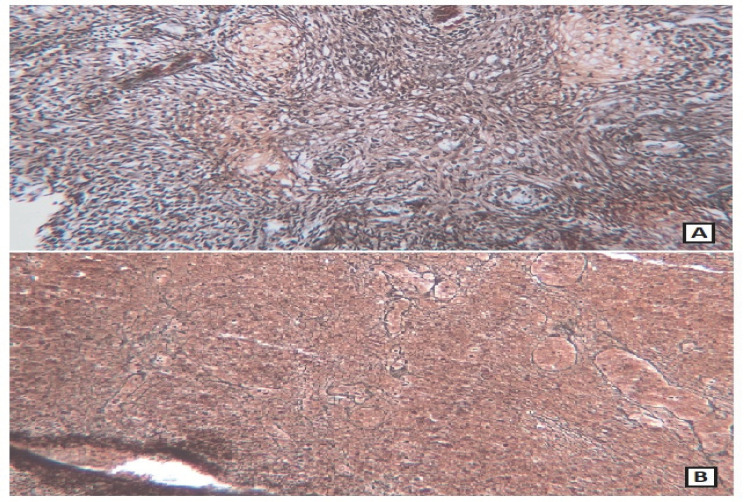
A: Diffuse reticulin fibers deposition in the sarcomatous region B: Absence of reticulin fibers in the glial region (Reticulin, 100× magnification)

**Fig. 6:**
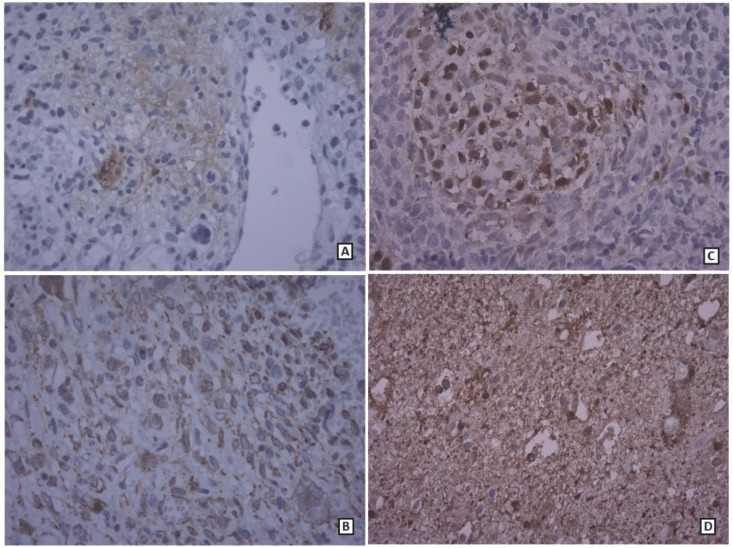
A: Immunohistochemical results: Glial region positive for GFAP B: Vimentin positive in GFAP negative mesenchymal component C: Positive S100 stain at chondrosarcomatous region D: Positive S100 stain at glial region, (400× magnification)

**Fig. 7:**
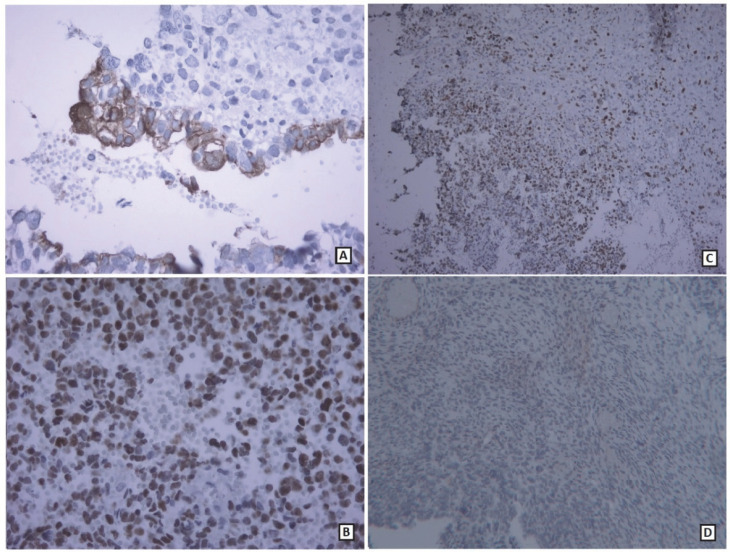
A: Immunohistochemical results: EMA positive in adenoid component (400× magnification) B: P53 expression in tumoral cells (400× magnification) C: Ki-67 expression in tumoral cells (100× magnification) D: Absence of IDH1 expression in glial and mesenchymal components (100× magnification)

Reticulin stain showed increased deposition of collagen within the mesenchymal areas (Figure 5). On immunohistochemistry, the glial component was GFAP positive. The mesenchymal component was positive for vimentin and negative for GFAP. S100 was positive in both glial and mesenchymal components (Figure 6). EMA showed positive reaction in the adenoid epithelial component. IDH1 had negative result in both glial and mesenchymal components. P53 and Ki-67 show 50% and 30% reaction in tumor cells, respectively (Figure 7).

On the second day of surgery, cardiac arrest happened and the patient died.

## Discussion

Gliosarcoma was first described by Stroebe in 1895 as a brain neoplasm consisting of both glial and mesenchymal components (3). It is defined as a variant of glioblastoma characterized by a biphasic tissue pattern with alternating areas displaying glial and mesenchymal differentiation (2). According to the World Health Organization classification, primary gliosarcoma (PGS) is considered to be a grade IV neoplasm, which is defined as a well-circumscribed lesion (4, 5).

Histologically, the glial component meets the cytologic criteria of glioblastoma, while the mesenchymal component may show a wide range of morphologies with origins ranging from fibroblastic, cartilaginous, osseous, smooth, and striated muscle, to adipose cell lineage (6). Despite the bi-phasic histology, genetic analyses suggest not only a molecular profile similar to glioblastoma, but also a monoclonal histogenesis for the glial and sarcomatous elements. Early reports have suggested that the sarcomatous component originates from the neoplastic transformation of hyperplastic blood vessels commonly observed in high grade gliomas (7, 8).

Another theory has recently drawn attention to a monoclonal origin for both components of gliosarcoma, with the sarcomatous component originating via aberrant mesenchymal differentiation of the malignant glioma. Recent studies have confirmed the argument that both elements of this biphasic neoplasm may arise from a single progenitor clone rather than separate clones. Both glial and mesenchymal elements may be derived from a common neoplastic neuroectodermal progenitor cell (6, 9, 10, 11). 

Vlodavsky* et al.* reported an extremely unusual variant of liposarcomatous differentiation in gliosarcoma displaying clusters of isolated lipoblasts in the sarcomatous portion of the tumor (12). Rajeshwari* et al.* reported a rare case of gliosarcoma with chondrosarcomatous differentiation, which can occur as one of the types of mesenchymal differentiation in this tumor (13). The study of Rodriguez* et al.* showed that a very uncommon morphological variation in high grade astrocytomas and also gliosarcomas was pseudoepithelial morphology. This mostly consists of an *adenoid* pattern mimicking adenocarcinoma, and less frequently, simply a large cell or *epithelioid* pattern. True epithelial differentiation in the form of squamous nests and true glands rarely occurs in gliosarcoma (14, 15).

In comparison to differential diagnosis such as immature teratoma and malignant intracerebral nerve sheath tumors findings of primitive neuroectodermal component and negative GFAP staining are helpful respectively (1, 16).

Given the available evidence, it is obvious that the occurrence of multiple types of sarcomatous differentiation in gliosarcoma is rare. We reported one case of tumor that presented with two different components as chondrosarcomatous and epithelial structures as adenoid formations, which is very rare and has not yet been reported. According to the IHC findings, which revealed negative expression of IDH1, similar to most forms of gliosarcoma, this case is also an IDH-wildtype form that supports its de novo nature.

## Conclusion

Even though gliosarcoma is a malignant growth with biphasic architecture of gliomatous and sarcomatous tissues**,** it may also have mesodermal-type elements such as adenoid formations.
